# Syndecans modulate ghrelin receptor signaling

**DOI:** 10.1530/JME-24-0070

**Published:** 2024-12-19

**Authors:** Karina Prins, Noa Mutsters, Femke Volker, Martin Huisman, Rosinda Mies, Patric J D Delhanty, Jenny A Visser

**Affiliations:** Department of Internal Medicine, Erasmus MC, University Medical Center Rotterdam, Rotterdam, The Netherlands

**Keywords:** ghrelin, GHSR, β-arrestin, G protein‐coupled receptor (GPCR), signal transduction

## Abstract

Ghrelin is a gut hormone that enhances food intake and growth hormone secretion through its G-protein coupled receptor, the growth hormone secretagogue receptor (GHSR). Recently, we have shown that ghrelin interacts with syndecans (SDCs), a family of membrane proteins known to modulate hypothalamic appetite signaling. Here, we investigated whether SDCs impact ghrelin signaling at GHSR by assessing ghrelin-induced intracellular Ca^2+^ mobilization (iCa^2+^) and inositol phosphate 1 (IP1) production in HEK293 cells. Compared with controls, the overexpression of SDCs dose-dependently increased the maximum iCa^2+^ response two- to four-fold, without affecting EC_50_. The IP1 response was similarly amplified by SDCs, but it also indicated that they reduce constitutive (ghrelin-independent) activity of GHSR. These enhanced responses occurred despite a SDC dose-dependent reduction in plasma membrane GHSR levels. Although ghrelin-stimulated Gα_q_ activation was unaltered by SDC1 expression, it failed to restore iCa^2+^ responsiveness in GNAQ/11 knockout cells, indicating dependence on Gα_q/11_, not another Gα subunit. This suggests that SDCs modulate either signaling downstream of Gα_q/11_ or quenching of β-arrestin2 recruitment to GHSR. Indeed, expression of SDCs at levels that only modestly suppress cell surface receptor reduced ghrelin-induced β-arrestin2 recruitment by ∼80%. SDC co-expression also delayed the peak β-arrestin2 response. However, peak β-arrestin2 recruitment follows the peak iCa^2+^ response, making it unclear whether reduced β-arrestin2 recruitment potentiated Ca^2+^ signaling. Altogether, SDCs enhanced iCa^2+^/IP1 and reduced β-arrestin2 recruitment by GHSR in response to ghrelin, likely by modulating signaling downstream of Gα_q_. This could be a novel mechanism through which SDCs affect metabolism and obesity.

## Introduction

Ghrelin is a peptide hormone that enhances appetite and regulates metabolism in energy-deficient states. Its acylated form (termed ‘ghrelin’ (Perello *et al.* 2023)) is the endogenous ligand of the growth hormone secretagogue receptor-1a (GHSR (Perello *et al.* 2023)). GHSR is mainly expressed in the brain: its expression in the hypothalamus regulates homeostatic eating and growth hormone secretion, whereas its presence in reward-related regions, such as the ventral tegmental area, is linked to reward signaling (Muller *et al.* 2015). GHSR is a G-protein coupled receptor (GPCR) that signals through Gα_q_. The activation of this signaling pathway rapidly increases cytosolic inositol-1,4,5-trisphosphate (IP3) levels, which acts on IP3 receptors on endoplasmic reticulum (ER) membranes to cause the release of intracellular Ca^2+^ from the ER. Following this, β-arrestin2 binds the intracellular tail of GHSR, blocking receptor interaction with Gα_q_ and quenching signaling. Moreover, β-arrestin2 coupling to GHSR designates the receptor for internalization (Cornejo *et al.* 2021). The role of GHSR, however, is not limited to ghrelin signaling. GHSR also dimerizes with other GPCRs, including dopamine (Kern *et al.* 2012), serotonin and oxytocin receptors, modulating their downstream signaling pathways (Price *et al.* 2021, Ringuet *et al.* 2022). Conversely, the function of GHSR itself is modulated by other membrane proteins, such as melanocortin receptor accessory protein 2 (MRAP2) (Srisai *et al.* 2017). MRAP2 reduces the constitutive activity of GHSR and enhances its activity upon ghrelin stimulation, as measured by the accumulation of inositol monophosphate, an IP3 metabolite (Rouault *et al.* 2020).

Recently, we have reported that both ghrelin and desacyl-ghrelin bind syndecan-1 (Delhanty *et al.* 2021). Syndecans (SDCs) are a group of four transmembrane heparan sulfate proteoglycans that consist of a variable glycosylated extracellular domain and share two conserved parts of their intracellular domains, flanking a variable intracellular region (Afratis *et al.* 2017). SDCs are widely expressed throughout the body, but in particular, SDC2 and SDC3 are expressed in the brain, including in the hypothalamic regions that regulate appetite. Interestingly, SDC3 knockout mice, as with ghrelin and GHSR knockout mice, are resistant to diet-induced obesity (Strader *et al.* 2004, Reizes *et al.* 2006). In humans, polymorphisms in SDC1 and SDC3 are associated with several anthropometric parameters related to obesity and, in cattle, with birth weight and body length (Ha *et al.* 2006, Schuring *et al.* 2009, Sun *et al.* 2011, Huang *et al.* 2016).

Considering the interaction between ghrelin and SDC1 and the involvement of SDCs in physiological processes that ghrelin also plays a role in, we aimed to investigate the effects of SDCs on ghrelin signaling. Here, using *in vitro* models to characterize ghrelin-induced Gα_q_ and calcium signaling, β-arrestin2 recruitment and cell surface expression, we show compelling evidence that SDCs modulate GHSR function.

## Experimental procedures

### Cell culture and transfection

HEK293 cells (ECACC 85120602, confirmed by karyotyping and STR PCR analysis), as well as the ΔGq/11 (CL1) GNAQ knockout and parental WT HEK293 cells (Schrage *et al.* 2015), were cultured in DMEM/F-12 (#31331, Gibco, The Netherlands), 10% heat-inactivated fetal calf serum (FCS; Gibco, The Netherlands) and 100 units/mL penicillin/100 µg/mL streptomycin (P/S; Gibco, The Netherlands). Transfections were performed using Fugene HD (Promega, The Netherlands) using a 1:3 DNA (μg):reagent (μL) ratio.

### SDC plasmids

pCMV3 vectors containing cDNAs for human SDC1 (NM_001006946.1), SDC2 (NM_002998.3), SDC4 (NM_002999.2) and negative control were obtained from SinoBiological (Germany). Because the SinoBiological SDC3 expression plasmid contained a 5′ truncated cDNA variant (SinoBiological, Germany; HG12158-UT; NCBI, BC013974), we generated a full-length pCMV3-SDC3 expression construct by inserting the correct 5′ region from a GenScript plasmid (GenScript, The Netherlands; Clone ID Ohu18173; NM_014654.4) utilizing *Hind*III to *Kfl*I restriction sites. All cDNAs were confirmed by Sanger sequencing.

### Aequorin assay

Mobilization of Ca^2+^ was measured by aequorin luminescence. The mitochondrial-targeted apo-aequorin (mtAEQ) expression plasmid was from Dr L Barak (Duke University Medical Center, USA), and the pcDNA3.1 (+)-GHSR (human) expression plasmid was from the cDNA Resource Center (www.cdna.org). 1.3 × 10^6^ cells were seeded in T25 flasks. The next day, cells were transiently transfected with 2 μg mtAEQ, 50 ng GHSR expression vector and 1 μg SDC or control vectors (1:20 GHSR:SDC). In experiments where different ratios of SDC were tested, the amount of SDC plasmid was supplemented to 1 μg with control vector. After 24 h, the transfected cells were washed with PBS (Gibco, The Netherlands) and harvested with PBS containing 5 mM Na_2_EDTA (Sigma, The Netherlands). Cells were resuspended in DMEM (Gibco, The Netherlands; 31053), supplemented with 2 mM L-glutamine, 0.1% BSA (Sigma, The Netherlands) and P/S. Cells at 1 × 10^6^ cells/mL were then incubated in 5 µM coelenterazine-h (Cayman, USA) for 3 h on a roller bank at room temperature. Next, the cell suspension was diluted five-fold and incubated for 1 h. A three-fold dilution range of ghrelin was prepared in white-bottom 96-well plates. Immediately after injecting ±1 × 10^4^ cells in a well, the ghrelin response was measured as luminescent signal in a plate reader (Victor X4; Perkin Elmer, The Netherlands, for dose–response curves; CLARIOstar Plus, BMG LabTech, Germany, for rapid kinetics, read luminescence every second). To account for the total activity of aequorin in the well, the remaining luminescence upon lysis with 0.1% Triton X-100 (Sigma, The Netherlands) was measured. For dose–response curves, the area under the curve of the luminescent responses was used. The ghrelin response was corrected for total luminescence (i.e., the sum of both ghrelin and triton measurements), yielding the fractional response (Delhanty *et al.* 2010).

### Inositol phosphate 1 (IP1) assay

HEK293 cells were seeded into T25 flasks (1.3 × 10^6^ cells/flask) and, the following day, were transiently co-transfected with GHSR and either a control (pCMV3) or SDC1 plasmids at 1:1 and 1:20 ratios using Fugene HD as for the aequorin assay. The total amount of plasmid mix transfected was corrected to 1 μg with control plasmid; 48 h later, the cells were dissociated with TrypLE Express (Thermo Fisher, The Netherlands), resuspended in stimulation buffer B (IP-One Gq HTRF kit; Perkin Elmer, The Netherlands) and distributed at 40,000 cells/well into a low-volume, opaque white homogeneous time-resolved fluorescence (HTRF) plate (Cisbio, The Netherlands). The cells were then treated for 1 h at 37°C with a range of ghrelin doses. Subsequently, total IP1 levels were assayed using the IP-One Gq HTRF kit according to the manufacturer’s instructions. Fluorescence intensities were measured at 665 and 620 nm using a CLARIOstar Plus microplate reader (BMG LabTech, Germany). The absolute IP1 concentrations were interpolated from a standard curve using the 665/620 ratio, and data were normalized either to baseline IP1 levels for each dose–response curve or to Emax of the control condition (no SDC1).

### GHSR localization and internalization

The localization of GHSR at the plasma membrane was investigated using the HiBiT Extracellular Detection System (Promega, The Netherlands). To N-terminally tag GHSR with HiBiT, its cDNA was PCR amplified from pcDNA3.1-GHSR using Phusion turbo polymerase (Thermo Fisher Scientific, The Netherlands), ligated into pBiT3.1-secN (Promega, The Netherlands) and confirmed by Sanger sequencing. For the assay, cells were seeded into the wells of white clear-bottom 96-well plates (Greiner Bio-One, Austria) and, after 24 h, each well was transfected using Fugene HD with 0.25 ng of HiBiT-GHSR plasmid and either SDC1, ranging from 25 pg to 5 ng (GHSR:SDC1 ratios of 1:0.1, 1:5, 1:10 and 1:20), or MRAP2 (GHSR:MRAP2, 1:20) expression plasmids. The total amount of DNA transfected was equalized using the transfection carrier DNA provided in the kit (Promega, The Netherlands). The following day, the medium was removed and replaced with 50 μL CO_2_-independent medium, 0.5% FCS. Fifty microliters of LgBiT protein and HiBiT extracellular substrate (diluted 1:100 and 1:50, respectively, in HiBiT extracellular buffer) were then added to each well. The plate was incubated for 8 min in a CLARIOstar Plus plate reader (BMG LabTech, Germany), and then luminescence was measured. To assess ghrelin-induced internalization, ghrelin (final concentration 1 μM) or vehicle was added to the wells and luminescence was monitored every 2 min for 20 min. Internalization data were normalized to baseline at the time of ligand/vehicle addition and then expressed as percent response relative to vehicle.

Bystander BRET experiments were performed using a modification of a previously described method (Wan *et al.* 2018). Expression plasmids encoding GHSR tagged at the N-terminus with NanoLuc luciferase (GHSR-NLuc) (kindly provided by Dr C Gorvin, University of Birmingham, UK) and mVenus-tagged CAAX motif derived from KRas (kindly provided by Dr N Lambert, Augusta University, USA) were combined (50 ng each) with either SDC (GHSR:SDC ratios of 1:0, 1:0.1, 1:5, 1:10 and 1:20) or MRAP2 (GHSR:MRAP2, 1:20) expression plasmids. If necessary, the total amount of plasmid was equalized with control plasmid. The plasmid mixtures were then transfected using Fugene HD into 210,000 cells in suspension and distributed into poly-D-lysine-coated clear-bottom white 96-well plates at 30,000 cells/well. After 48 h, the cells were washed with Hank's balanced salt solution (HBSS), which was then replaced with 50 μL/well of NanoBRET reagent (Promega, The Netherlands; diluted 1:1,000 in HBSS). Ten minutes later, emission intensities were read at 450 ± 20 nm (NLuc luminescence) and 530 ± 20 nm (mVenus fluorescence) in a CLARIOstar Plus plate reader (BMG LabTech, Germany). netBRET, a measure of cell surface receptor levels, was calculated as ((fluorescence at 530 ± 20 nm/luminescence at 450 ± 20 nm)) − (background (fluorescence at 530 ± 20 nm/luminescence at 450 ± 20 nm)), with the background corresponding to the signal produced by cells expressing GHSR-NLuc alone under similar conditions. Total levels of GHSR expression were derived from the GHSR-NLuc luminescence signal directly.

### Gα_q_ activation

Gα_q_ subunit activation upon ghrelin stimulation was determined using a Gα_q_ bioluminescence resonance energy transfer (BRET) biosensor (Gq-CASE was a gift from Gunnar Schulte: Addgene plasmid #168125; https://www.addgene.org/168125/; RRID: Addgene_168125). 3 × 10^5^ HEK293 cells were transfected in 1 mL suspension with 500 ng Gq-CASE; 100 ng pcDNA3.1 (+)-GHSR; and 0, 10, 100, 500 and 1,000 ng SDC1 expression plasmid (GHSR:SDC ratios of 1:0, 1:0.1, 1:1, 1:5 and 1:10). The amount of plasmid transfected was equalized with control vector. Cells were then seeded at 3 × 10^4^ cells per well in PDL-coated opaque white 96-well plates. After 48 h, cells were loaded with furimazine (Promega, The Netherlands) in HBSS (Gibco) and stimulated with a dose curve of ghrelin ranging from 10^−12^ to 10^−6^ M. The BRET signal was measured 3 min following the addition of ghrelin. For kinetics experiments, each well was assessed individually. Baseline BRET was measured for 4 s, then ghrelin was injected, and the BRET signal was measured every second following treatment. Data from the first 60 s were analyzed. Emission intensities for cpVenus and Nluc were measured at wavelengths of 535 ± 30 nm and 450 ± 40 nm, respectively, in a CLARIOstar Plus plate reader with an injector (BMG LabTech, Germany). The BRET ratio (cpVenus/Nluc) declines upon activation of Gα_q_. To quantify ligand-induced changes, ΔBRET was calculated for each well as a percent over basal (((ratio_stim_ − ratio_basal_)/ratio_basal_) × 100) and then the average ΔBRET of vehicle control was subtracted (Schihada *et al.* 2021) giving ΔBRET%.

### β-arrestin2 recruitment

GHSR and human β-arrestin2 cDNAs were cloned into NanoBiT vectors (Promega, The Netherlands). GHSR was tagged with C-terminal SmBiT using pBiT2.1-C (GHSR-SmC), and β-arrestin2 was N-terminally labeled with LgBiT using pBiT1.1-N (β-arrestin2-LgN). Plasmids were confirmed by Sanger sequencing. HEK293 cells were transfected with 50 ng GHSR-SmBiT and 50 ng LgBiT-β-arrestin2 and varying amounts (25, 50 and 100 ng) of SDC plasmid (GHSR:SDC ratios of 1:0.5, 1:1 and 1:2). After 24 h, culture medium was replaced with CO_2_-independent medium (#18045054; Gibco, The Netherlands), supplemented with 0.5% FCS, and 1× Nano-Glo substrate (Promega, The Netherlands) was added. Luminescence was measured in a plate reader (CLARIOstar Plus; BMG LabTech, Germany); after baseline luminescence measurements, the ghrelin response of the cells was measured for 20 min. The ghrelin response was expressed as the fold-change over baseline.

### Data analysis

Data were imported into Prism 10.0 (GraphPad, USA; www.graphpad.com), in which graphs and dose–response curves were generated. Curve-fitting and derivation of kinetic parameters from time-course experiments were performed using plugins for Prism (‘baseline then rise and fall to steady-state’ algorithm for β-arrestin2 recruitment data and ‘baseline then fall to steady state’ for Gq-CASE response data, Pharmechanics, USA). Kinetics data for β-arrestin2 recruitment were also analyzed by repeated-measures two-way ANOVA, followed by post hoc analyses. For dose–response curves, maximum response (Emax), EC_50_ and baseline were analyzed by ANOVA, followed by Tukey’s post hoc testing. The effect of MRAP2 on cell surface GHSR expression was assessed by t-test. Mean ± SEM are reported, and differences were considered significant if *P* < 0.05.

Single-cell RNA sequencing data (GEO accession GSE74672 (Romanov *et al.* 2017)) were interrogated for co-expression in single hypothalamic neurons of *Ghsr* with *Sdc1-4* or *Mrap2*. *Ghsr*-expressing cells were selected, and SDCs and *Mrap2* in these cells was expressed as the number of RNA molecules/10,000 RNA molecules per cell.

## Results

### SDCs dose-dependently enhance ghrelin-stimulated Ca^2+^ signaling

**Figure 1:**
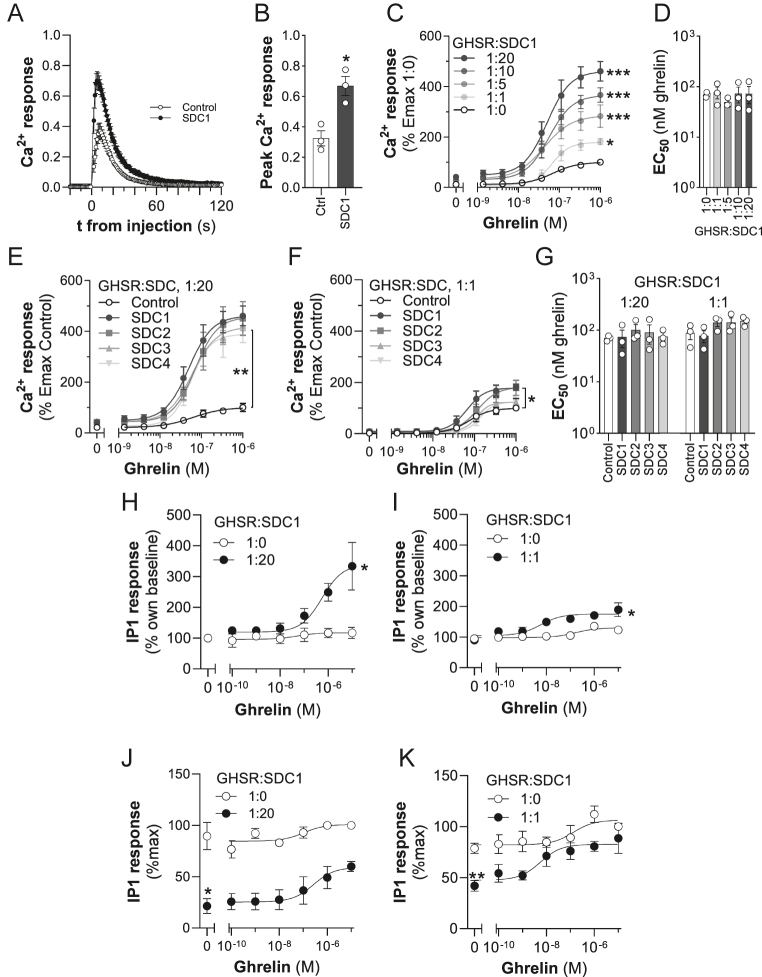
Syndecans (SDCs) enhance ghrelin-stimulated growth hormone secretagogue receptor (GHSR) signaling. (A) Time course of aequorin luminescence after ghrelin treatment in cells expressing GHSR with control or SDC1 expression plasmids at 1:20 ratio. (B) Comparison of peak iCa^2+^ responses derived from curve-fitting data from A (*P < 0.05, GHSR alone versus SDC1 co-transfection). (C) Dose–response curves of intracellular ghrelin-stimulated iCa^2+^ expressed as % Emax of 1:0 ratio: effect of different GHSR:SDC1 transfection ratios (*P < 0.05, ***P < 0.0005, Emax for GHSR:SDC co-expression versus GHSR alone (GHSR:SDC, 1:0) tested by ANOVA). (D) EC_50_s for ghrelin derived from dose–response curves shown in (C) (SDC1 ratios). (E) Effect of all SDCs co-transfected at a GHSR:SDC ratio of 1:20 (**P < 0.005 (t-test), Emax for GHSR:SDC co-expression versus GHSR alone). (F) Effect of all SDCs co-transfected at a GHSR:SDC ratio of 1:1 (*P ≤ 0.05 (t-test), Emax for GHSR:SDC co-expression versus GHSR alone). (G) EC_50_s for ghrelin derived from SDC1–SDC4 dose–response curves shown in (E) and (F). (H) and (I) Dose–response curves of IP1 accumulation after ghrelin stimulation of cells expressing GHSR in the presence or absence of SDC1 at 1:20 and 1:1 ratios, respectively, corrected for baseline (*P ≤ 0.05 (t-test), Emax ghrelin-induced IP1 levels of 1:0 v. 1:20 or 1:1 ratio). (J) and (K) Dose–response curves of IP1 levels after ghrelin stimulation of cells expressing GHSR in the presence or absence of SDC1 at 1:20 and 1:1 ratios, respectively, expressed as % of maximum response in the absence of SDC1 (*P ≤ 0.05, **P < 0.01 (t-test), basal IP1 levels of 1:0 v. 1:20 or 1:1 ratio, respectively). Data are mean ± SEM of three or more independent experiments.

We investigated the effect of SDCs on ghrelin-stimulated Gα_q_ signaling by measuring intracellular Ca^2+^ mobilization (iCa^2+^) using an aequorin-based assay. This assay enables the measurement of rapid increases in iCa^2+^ in live cells within seconds of ghrelin stimulation in HEK293 cells (Fig. 1A). We found that following ghrelin treatment, the peak iCa^2+^ response was significantly increased (*P* < 0.05; Fig. 1B). The peak response time was also significantly decreased in SDC1 transfected cells relative to controls from 7.7 ± 0.7 s to 5.6 ± 0.3 s for control and SDC1 co-transfected cells, respectively (*P* < 0.05). These altered kinetics suggest that SDC1 could modulate the amount of stored Ca^2+^, the rate of depletion of Ca^2+^ stored and/or the rate of clearance of cytoplasmic Ca^2+^ out of the cell (Hoare *et al.* 2020). Co-transfection of GHSR with different amounts of SDC1 expression plasmid (GHSR:SDC1 of 1:0 to 1:20) dose-dependently increased the Emax of the iCa^2+^ response upon ghrelin stimulation, with the highest GHSR:SDC1 ratio (1:20) resulting in a 510 ± 90% increase relative to controls (Fig. 1C). However, neither the EC_50_ (Fig. 1D) nor the basal response was affected by SDC1 expression. Co-transfection of SDC2–SDC4 at the GHSR:SDC ratio of 1:20 enhanced the maximum iCa^2+^ response similarly to SDC1 (*P* ≤ 0.005 for all SDCs vs control) (Fig. 1E). At the 1:20 ratio, all 4 SDCs behaved similarly, increasing the efficacy of ghrelin in stimulating iCa^2+^, but having no effect on the potency of ghrelin (Fig. 1G). However, at a lower 1:1 ratio, only SDC1 and SDC2 caused a significant (∼200%, *P* ≤ 0.05) potentiation of the ghrelin-induced iCa^2+^ maximal response (Fig. 1F), although again, the potency of ghrelin remained unaffected (Fig. 1G).

Many GPCRs are now recognized to exhibit constitutive, ligand-independent, activity. However, iCa^2+^ is not a sensitive method to detect such basal activity because cellular Ca^2+^ levels are stringently regulated and therefore responses are extremely transient (Holst *et al.* 2003). To overcome this, IP3 production was used to identify the constitutive activity of GHSR (Holst *et al.* 2003) and, relevant to this study, MRAP2 was found to markedly suppress basal GHSR activity as assessed by IP1 accumulation (Rouault *et al.* 2022). To assess whether, like MRAP2, SDC1 modulated the constitutive activity of GHSR, we also used IP1 as a readout for its ligand-induced and constitutive activity. Like iCa^2+^, the Emax of IP1 was potentiated significantly by SDC1 to 3-fold (1:20 GHSR:SDC1 ratio, *P* < 0.05) and 1.5-fold (1:1 GHSR:SDC1 ratio, *P* < 0.05) of controls when data were expressed relative to baseline (Fig. 1H and I). Moreover, like MRAP2, SDC1 significantly suppressed constitutive, ligand-independent, GHSR-mediated activity while intensifying the ghrelin-stimulated response at both 1:20 and 1:1 GHSR:SDC1 ratios (Fig. 1J and K).

### SDCs dose-dependently suppress GHSR levels at the plasma membrane

**Figure 2:**
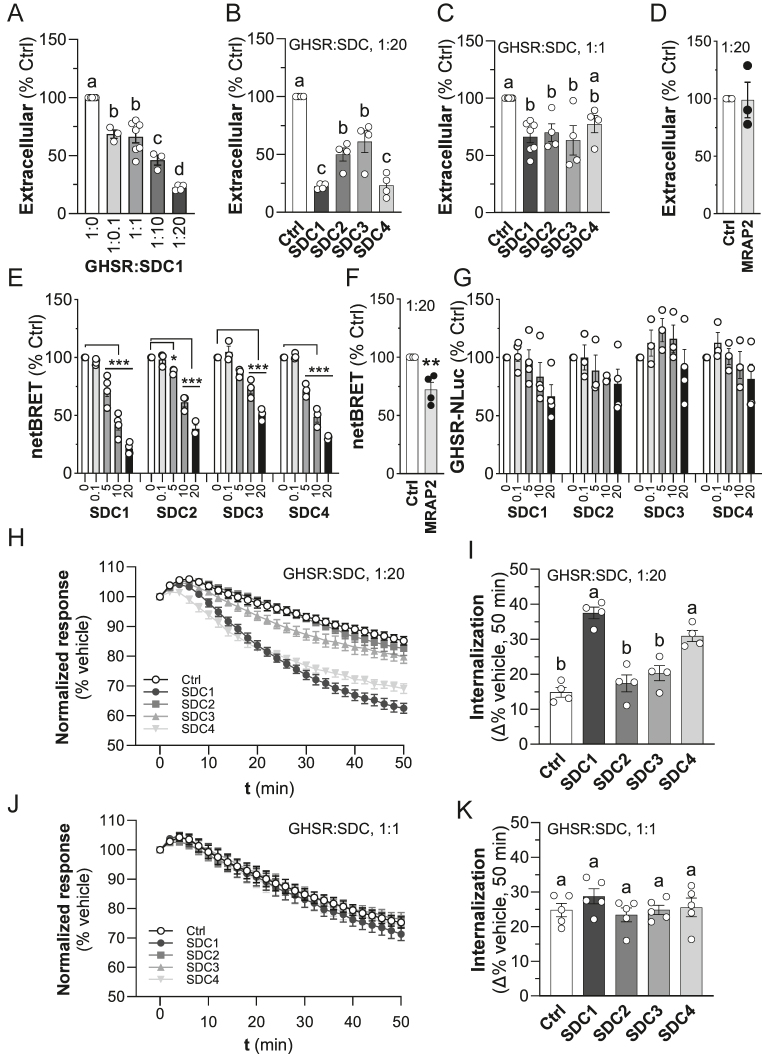
Syndecans (SDCs) reduce plasma membrane growth hormone secretagogue receptor (GHSR) availability. Plasma membrane (extracellular) expression of GHSR-HiBiT. (A) Effect of increasing GHSR:SDC1 expression plasmid ratios on cell surface levels expressed as % luminescence intensity relative to control (1:0 ratio). (B) Effect of all SDCs (GHSR:SDC, 1:20) expressed as % luminescence intensity relative to control (1:0 ratio). (C) Effect of all SDCs (GHSR:SDC, 1:1) expressed as % luminescence intensity relative to control (1:0 ratio). (D) Effect of MRAP2 on ghrelin-induced GHSR internalization using HiBiT. (E) Bystander BRET evaluation of effect of all SDCs on cell surface levels of GHSR expressed as netBRET % of 1:0 control (GHSR:SDC ratios of 1:0 to 1:20; *P < 0.05, ***P < 0.005 versus 1:0). (F) Effect of MRAP2 on ghrelin-induced GHSR internalization using bystander BRET. (G) Effect of SDCs on total GHSR expression assessed by measuring GHSR-NLuc derived luminescence of bystander BRET assays (**P < 0.01). (H) Effect of SDCs at a GHSR:SDC ratio of 1:20 on 1 μM ghrelin-induced internalization using the HiBiT assay. (I) Percentage internalization 50 min following ghrelin treatment relative to vehicle from (H). (J) Effect of SDCs at a GHSR:SDC ratio of 1:1 on 1 μM ghrelin-induced internalization using the HiBiT assay. (K) Percentage internalization 50 min following ghrelin treatment relative to vehicle from (J). Data are corrected for baseline and then expressed as % of vehicle controls at each time point. Data are presented as mean ± SEM of 3–7 independent experiments. Letters a–d represent groups that are significantly different from each other (*P* < 0.05; one-way ANOVA).

As SDCs and GHSR both localize to the plasma membrane, we hypothesized that SDCs might increase cell surface localization of GHSR as an explanation for the observed enhanced ghrelin response. In contrast to our hypothesis, we found that co-transfection of SDC1 dose-dependently reduced the GHSR cell surface (extracellular) expression, as assessed using a NanoBiT complementation assay (HiBiT, Fig. 2A). Transfection with the greatest GHSR:SDC1 expression plasmid ratio (1:20) caused a 78 ± 1% reduction in cell surface (extracellular) GHSR levels compared to controls (*P* < 0.0005). Similarly, SDC2, SDC3 and SDC4 when transfected at the 1:20 GHSR:SDC ratio all significantly reduced cell surface levels of GHSR by between 50 and 77%, compared to the control (*P* < 0.005; Fig. 2B). However, when transfected at a GHSR:SDC ratio of 1:1, cell surface levels were less affected, reducing GHSR cell surface levels by only 25–30% relative to controls (Fig. 2C). A possible explanation for the suppression of cell surface levels of the receptor is through a gene dosage effect, whereby co-expression of two genes, as opposed to one, drives down the production of both. MRAP2 has been shown to modulate GHSR signaling but only modestly suppress cell surface levels at a GHSR:MRAP2 ratio of 1:20, as assessed using a fixed-cell ELISA (Rouault *et al.* 2020). Therefore, we have evaluated a possible gene dosage effect in our HiBit complementation assays while co-expressing GHSR and MRAP2 at a ratio of 1:20. We observed no effect of MRAP2 (Fig. 2D). This suggests that there is little or no gene dosage effect on cell surface receptor levels.

To confirm this finding, we used bystander BRET with plasmids encoding the plasma membrane biosensor C-terminal KRas tagged with Venus as the acceptor and GHSR tagged with NLuc as the donor (Lan *et al.* 2012). The effect of SDCs on cell surface GHSR measured using this method is broadly similar to those obtained with HiBiT (Fig. 2E). Using this assay, we also observed a modest ∼25% (*P* < 0.01) suppression of cell surface GHSR levels by MRAP2 (Fig. 2F). Bystander BRET has the advantage that the luminescent signal from GHSR-NLuc is assessed at the same time as BRET-induced fluorescence in the same cells and can be used to assess the effect of SDCs on total levels of GHSR expression. We found that total levels of GHSR-NLuc expression are not significantly affected by SDC overexpression (Fig. 2G), indicating that cell surface expression of GHSR by SDCs is modulated independent of its overall expression and further suggests a lack of a gene dosage effect.

Despite having almost identical effects on the potentiation of ghrelin-induced iCa^2+^, at a GHSR:SDC ratio of 1:20, the SDCs had differential effects on rates of ligand-induced cellular internalization (Fig. 2H). Ghrelin-induced internalization was significantly greater compared to empty vector controls in the presence of SDC1 and SDC4, but not in the presence of SDC2 and SDC3 (Fig. 2I). However, at a GHSR:SDC ratio of 1:1, this differential effect on internalization was lost (Fig. 2J and K).

### SDCs require GHSR-mediated Gα_q_ activation to potentiate the ghrelin-induced Ca^2+^ response

**Figure 3:**
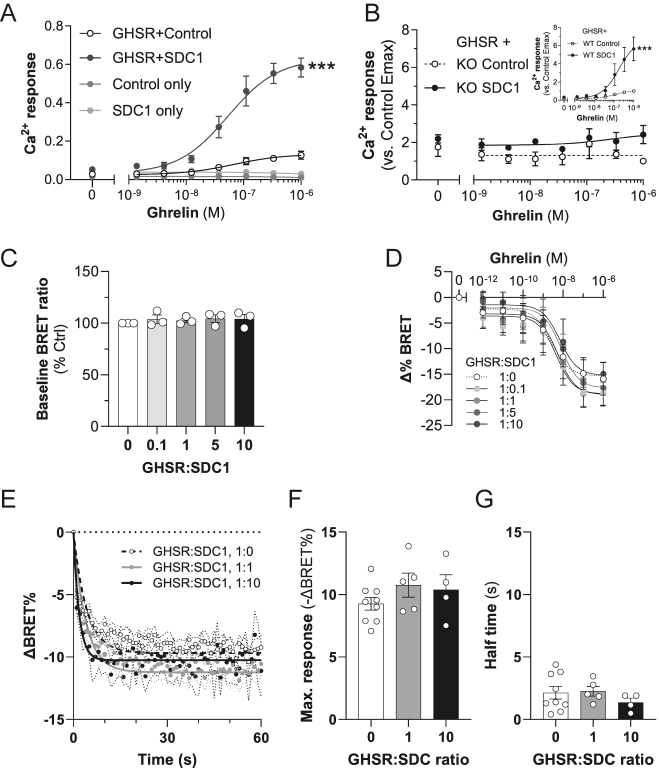
Syndecans (SDCs) require growth hormone secretagogue receptor (GHSR) and Gα_q/11_ to enhance ghrelin-induced signaling but do not potentiate activation of Gq directly. (A) Effect of SDC1 on ghrelin-stimulated intracellular calcium levels in cells with or without GHSR expression. (B) Effect of SDC1 on ghrelin-stimulated Ca^2+^ release in GNAQ/11 WT (inset) and KO HEK293 cells (***P < 0.0005 versus own control. WT, wild-type parental cell-line; KO, GNAQ/11 knockout. Data are corrected for WT control Emax). (C) Lack of effect of different GHSR:SDC1 ratios on ligand-independent Gα_q_ biosensor activity. (D) Lack of effect of different GHSR:SDC1 ratios on ghrelin-stimulated Gα_q_ activation, as measured by a decrease in BRET over the prestimulation baseline. (E) Kinetics of the Gq-CASE response during the first 60 s following ghrelin treatment, showing 1:0 (control), 1:1 and 1:10 GHSR:SDC1 ratios (SEM represented by the dotted lines). (F) and (G) Maximal response and half-times derived from curve-fitting of data shown in (E) (ANOVA gave no significant differences). For all experiments, data are presented as mean ± SEM of 3–4 independent experiments.

Because the potentiating effect of SDC overexpression on the ghrelin-induced iCa^2+^ response contrasts with a concomitant reduction in GHSR cell surface expression, we investigated whether SDCs could be independent receptors for ghrelin. We analyzed this by assessing ghrelin-induced iCa^2+^ in the absence of GHSR with or without SDC1 (Fig. 3A). However, ghrelin did not evoke an iCa^2+^ response in cells transfected with SDC1 alone. Only upon co-transfection of both GHSR and SDC1 was the potentiation of the ghrelin-induced iCa^2+^ response observed (GHSR + Ctrl vs GHSR + SDC1; plasmid ratio of 1:20, *P* < 0.0001; Fig. 3A). This demonstrated that the effect of SDC1 on ghrelin-induced intracellular Ca^2+^ mobilization requires GHSR.

To determine whether the SDC-induced potentiation of the iCa^2+^ response required Gα_q_-dependent signaling, we used GNAQ/11-knockout HEK293 cells (Schrage *et al.* 2015) transfected with GHSR. The absence of Gα_q/11_ abolished the ghrelin-induced Ca^2+^ response of the cells both in the presence and absence of SDC1 (Fig. 3B). In the WT parental cell-line, SDC1 potentiated ghrelin-induced iCa^2+^ as normal (Fig. 3B, inset). These observations indicate that Gα_q/11_, and not another Gα protein subtype, mediates the potentiation of ghrelin-induced Ca^2+^ signaling measured in the aequorin assay. Therefore, we next analyzed whether SDCs affect Gα_q_ activation in GHSR-transfected cells, using Gq-CASE, a BRET-based biosensor of Gα_q_ activity (Schihada *et al.* 2021). Interestingly, none of the GHSR:SDC1 expression plasmid ratios (1:0 to 1:10) we transfected affected the constitutive or ghrelin-induced Gα_q/11_ activation (Fig. 3C and D). We then tested the effect of GHSR:SDC1 ratio on the kinetics of the Gq-CASE response during the first 60 s following ghrelin treatment (Fig. 3E). The timing of the response corresponded well with the iCa^2+^ response time, reaching a maximal effect within ∼5 s of treatment with ghrelin. However, we found that SDC1 had no significant effect on either the maximum BRET response or the half-time of the response at GHSR:SDC1 ratios of either 1:1 or 1:10 (Fig. 3F and G). These results suggest that the effect of SDCs on ghrelin-induced intracellular Ca^2+^ signaling requires GHSR-mediated Gα_q/11_ activation, but that SDCs do not directly modulate Gα_q_ function.

### SDCs inhibit β-arrestin2 recruitment to GHSR

**Figure 4:**
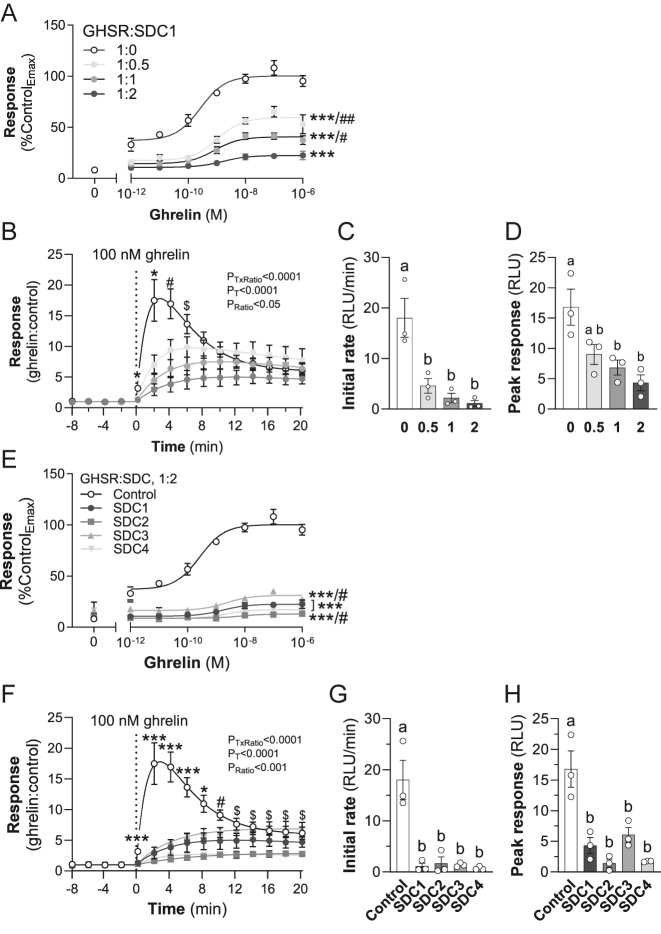
Syndecans (SDCs) impair β-arrestin2 recruitment at growth hormone secretagogue receptor (GHSR). (A) Effect of SDC1 on ghrelin-stimulated β-arrestin2–GHSR interaction in HEK293 cells. Values were corrected for Emax of the control (***P < 0.0005 versus 1:0 ratio GHSR:SDC1; ^#^ *P* < 0.05, ^##^ *P* < 0.005 versus 1:2 ratio GHSR:SDC1). (B) Effect of SDC1 on the kinetic profile of the β-arrestin2–GHSR interaction upon ghrelin stimulation (outcome of RM-ANOVA shown on graph: *P*_T_, effect of time; *P*_Ratio_, effect of GHSR:SDC ratio; *P*_TxRatio_, interaction. *P < 0.05, all other ratios versus 1:0 ratio GHSR:SDC1; ^#^ *P* < 0.05, 1:1 and 1:2 versus 1:0 ratio; ^$^ *P* < 0.05, 1:2 versus 1:0 ratio). Data derived from curve fitting of the kinetic profiles for β-arrestin2 recruitment by GHSR in cells transfected with control or increasing amounts of SDC1 expression plasmid. (C) Initial rate of recruitment. (D) Magnitude of peak response (letters a and b represent groups that are significantly different from each other (*P* < 0.05; one-way ANOVA). Dose–response curve (E) (***P < 0.0005 versus control; ^#^ *P* < 0.05 versus 1:2 ratio GHSR:SDC1) and kinetic curve (F) of the effect of all SDCs (GHSR:SDC of 1:2) on β-arrestin2 recruitment at GHSR (outcome of RM-ANOVA shown on graph. *P < 0.05, ***P < 0.001, all SDCs versus control; ^#^ *P* < 0.05, SDC1, SDC2 and SDC4 versus control; ^$^ *P* < 0.05, SDC2 and SDC4 versus control. *P*_T_, effect of time; *P*_Ratio_, effect of GHSR:SDC ratio; *P*_TxRatio_, interaction). Data derived from curve fitting of the kinetic profiles for β-arrestin2 recruitment by GHSR in cells transfected with control or SDC1, SDC2, SDC3 or SDC4 expression plasmids. (G) Initial rate of recruitment. (H) Magnitude of peak response (letters a and b represent groups that are significantly different from each other (*P* < 0.05; one-way ANOVA). Data are presented as mean ± SEM of three independent experiments. Emax, maximum response.

As overexpression of SDCs did not affect the activation of Gα_q_, we investigated whether the quenching of GHSR signaling was altered in the presence of SDCs. For this purpose, we investigated β-arrestin2 recruitment to GHSR, using a NanoBiT complementation assay. Because SDCs markedly suppress cell surface levels of GHSR when the expression plasmids were co-transfected at GHSR:SDC ratios greater than 1:5, we assessed plasmid ratios below this threshold (1:0.5, 1:1 and 1:2). This meant that β-arrestin2 recruitment levels could be assessed when cell surface receptor expression levels are only modestly affected. Co-transfection of SDC1 markedly and dose-dependently inhibited ghrelin-stimulated maximal β-arrestin2 recruitment to GHSR by 40–77% (ANOVA *P* < 0.0001; Fig. 4A).

We next examined the kinetics of ghrelin-induced β-arrestin2 recruitment by GHSR using a curve-fitting algorithm (Fig. 4B). In the absence of SDC1, the initial rate of recruitment of β-arrestin2 by GHSR is relatively rapid (18 ± 3.8 RLU/min) and reaches a peak of 16.8 ± 3 RLU at 2.9 ± 0.3 min following treatment with ghrelin (Fig. 4C and D). After reaching a peak, the luminescence/level of association between β-arrestin2 and GHSR declined to a steady state that plateaued at about 20 min. The presence of SDC1 significantly altered these kinetic parameters in a dose-dependent way, markedly suppressing the initial recruitment rate and peak response (Fig. 4C and D). The sharp rise in the GHSR:β-arrestin2 association and then a fall to a steady state seen in the absence of SDC1 was markedly blunted in the presence of SDC1, with a much reduced rate of decline to a steady state. This could indicate a distinct change in the mechanism of recruitment.

SDC2–SDC4 also reduced ghrelin-induced β-arrestin2 recruitment at GHSR (*P* < 0.0001; Fig. 4E) to a similar extent and with a similar kinetic profile as SDC1 (Fig. 4F). Analysis of these data demonstrated that all four SDCs caused significant reductions in initial rates and the peak level (response) of GHSR:β-arrestin2 association (Fig. 4G and H).

### Hypothalamic co-expression of SDCs and GHSR

**Figure 5:**
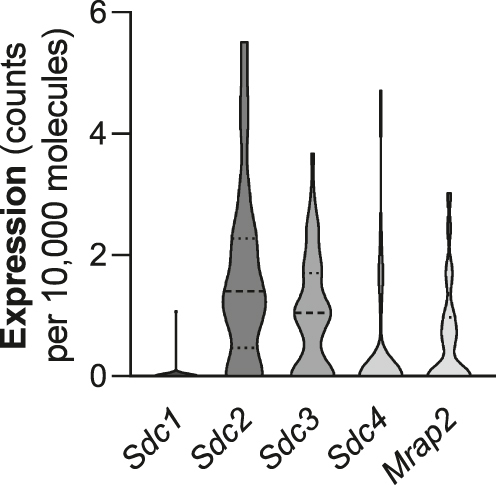
Syndecans (SDCs) are co-expressed with growth hormone secretagogue receptor (GHSR) in mouse hypothalamus. In a single-cell RNA-seq dataset, 47/50 cells that express GHSR also express one or more SDCs. Data are expressed as counts per 10,000 molecules.

To begin to assess the potential physiological relevance of our observations, we next analyzed a single-cell RNA sequencing dataset of the mouse hypothalamus to verify the co-expression of SDCs in GHSR neurons *in vivo* (GEO accession GSE74672 (Romanov *et al.* 2017)). Of the 50 cells expressing *Ghsr*, 47 expressed one or more of the SDCs (Fig. 5). In contrast, *Mrap2,* which encodes a membrane protein previously shown to modulate GHSR signaling (Srisai *et al.* 2017), was co-expressed with *Ghsr* in 24 of the 50 cells. *Sdc2* (median: 1.40 copies/10,000 molecules) and *Sdc3* (median: 1.05 copies/10,000 molecules) were most abundantly expressed.

## Discussion

In an earlier study, we found that SDC1 binds ghrelin via charge-dependent interactions with its heparan sulfate side chains (Delhanty *et al.* 2021). This finding led us to investigate whether SDCs could modulate ghrelin-induced GHSR signaling. Here, we show that SDC1 suppresses GHSR-dependent basal IP1 levels but intensifies the amplitude of the ghrelin-induced response, similar to the effect of MRAP2 (Rouault *et al.* 2020). Our data suggest that this translates to amplification of ghrelin-induced iCa^2+^ in GHSR-expressing cells, and we hypothesize that SDCs cause increased sensitivity of GHSR neurons to ghrelin. Although the effects of SDCs on neuronal function remain to be explored, these findings mean that the cellular context could be important for GHSR activity, as suggested by Rouault and coworkers (Rouault *et al.* 2020). GHSR may produce different signals dependent on the levels of expression of MRAP2 and/or SDCs in neurons in which it is expressed.

These effects of SDCs occur despite the partial suppression of receptor cell surface levels. We hypothesize that this is due to spare receptors in the overexpression system, where only a small percentage of available receptors is required for full efficacy of ligand. This phenomenon has been described for other GPCRs (Guieu *et al.* 2021). Furthermore, the effects of SDCs are dose dependent, and at GHSR:SDC ratios that only moderately suppress cell surface expression, significant 1.5- to 2-fold effects on maximal ghrelin responses (iCa^2+^ and IP1) are still observed. This dose-dependency of effect was also observed in relation to the modulation of GHSR internalization by SDCs: SDC1 and SDC4 potentiated internalization when co-expressed at 1:20 but not at a 1:1 ratio. However, GHSR was still required to accomplish the ghrelin-induced Ca^2+^ response, which illustrates that SDCs are not a novel ghrelin receptor by themselves but rather affect the activation of and signal transduction through GHSR. This effect was also Gα_q/11_ dependent since GNAQ/11-deficient HEK293 cells transfected with GHSR were unresponsive to ghrelin treatment using iCa^2+^ as a readout. SDC1 and SDC4 were also found to amplify the GHSR internalization rate. SDC4 has been reported to interact with and modulate the activity of protein kinase C-α (PKCα) (Keum *et al.* 2004). This could be a mechanism by which SDC4 alters ligand-induced GHSR internalization observed in this study, since PKCα has been shown to regulate phosphorylation and trafficking of other GPCRs (Namkung & Sibley 2004). Although SDC1 and SDC4 have been shown to interact directly with a range of kinase receptors and modulate their internalization (Afratis *et al.* 2017, Kang *et al.* 2020), the mechanism by which either of these SDCs could modulate GPCR internalization remains to be investigated.

Like MRAP2 (Rouault *et al.* 2020), we found that SDCs profoundly suppress ligand-induced β-arrestin2 recruitment to GHSR. This occurs with GHSR:SDC plasmid transfection ratios that only modestly affect receptor cell surface levels. Initially, we thought that this could be the mechanism behind our observation that SDCs potentiate the ligand-induced iCa^2+^ response. However, the enhancement of Ca^2+^ mobilization precedes peak β-arrestin2 recruitment by several minutes. Consequently, we think that modulation of β-arrestin2 recruitment to GHSR by SDCs does not explain the effect on iCa^2+^. Mechanistically, this appears to be similar to MRAP2, which retains its ability to potentiate a ligand-induced IP1 response from GHSR in cells that lack β-arrestin1 and β-arrestin2 (Rouault *et al.* 2020).

GPCRs are grouped into two classes based on the way they interact with β-arrestins and become internalized. Following ligand-induced recruitment, class A GPCRs interact only transiently with β-arrestins and rapidly dissociate from them at or near the plasma membrane, whereas class B GPCRs remain in stable complexes that are trafficked into the cell on endosomal vesicles (Spillmann *et al.* 2020). GHSR has been characterized as a class A GPCR since it lacks stable interaction with β-arrestins (Evron *et al.* 2014). This can also be seen in the kinetic profiles in our study in which ligand-induced association with β-arrestin2 reaches a peak that then declines to a lower steady state. SDCs, however, markedly change this kinetic profile, suppressing the early peak of recruitment, but prolonging the period of association with β-arrestin2. This suggests that the presence of SDCs causes a transition from a type A-like pattern of rapid receptor recycling to the plasma membrane to a more class B-like interaction characterized by a slower rise to a steady state, suggesting prolonged binding and subsequent internalization of the receptor.

While both SDCs and MRAP2 inhibit β-arrestin2 recruitment, the kinetics of the recruitment profile differs between these membrane proteins. In the presence of MRAP2, the kinetics of β-arrestin2 recruitment by GHSR is comparable to that of cells expressing GHSR only, albeit with a lower peak recruitment (Rouault *et al.* 2020, Rouault *et al.* 2022). However, we do not find a marked modulatory effect of SDCs on the rate of receptor internalization. It thus remains to be determined whether the effect of SDCs on GHSR:β-arrestin2 recruitment has other effects, such as impacting β-arrestin2 downstream signaling. Indeed, the regulation of β-arrestin2 recruitment and activity is known to be multidimensional, being affected by biased ligands, the type of GPCR and the phosphorylation profile of the GPCR (Chen *et al.* 2022). The latter, for example, was affected by MRAP2, resulting in the decreased β-arrestin2 recruitment to GHSR in its presence (Rouault *et al.* 2022). It could well be, however, that SDCs impact the interaction between GHSR and β-arrestin2 in a different way, due to our findings in the kinetics experiments. Although further studies are needed, our results do suggest that SDCs, like MRAP2, alter β-arrestin2 function in interaction with GPCRs.

Since reduced β-arrestin2 recruitment likely does not explain the potentiation by SDC1 of the ghrelin-induced iCa^2+^ response, the Gα_q_ signaling cascade itself may hold the answer. Furthermore, our results suggest that SDC-induced potentiation of ghrelin signaling was specific to Gα_q_ signaling because GHSR-transfected Gα_q/11_-deficient cells do not show an iCa^2+^ response to ghrelin. Neither the constitutive activity nor the ghrelin-induced activation of a Gα_q_ biosensor by GHSR was affected by SDC co-expression, suggesting that SDCs modulate GHSR signaling downstream of Gα_q_.

An important mechanism that cells utilize to rapidly elevate cytosolic and mitochondrial Ca^2+^ is through its mobilization from the ER. Activation of Gα_q_-coupled receptors stimulates phospholipase Cβ (PLCβ) to generate IP3, which then binds to IP3R in the ER membrane causing Ca^2+^ to flow out of stores in the ER (Kamato *et al.* 2017). This suggests two possible targets through which SDCs could affect the rapid effects on iCa^2+^ that we have observed: PLC and IP3R. Although there is no yet clear evidence for a direct effect of SDCs on PLC function, SDC4 has been shown, independently of the presence of a receptor, to bind PIP2 with high affinity and retain it in the plasma membrane, thus suppressing its rate of hydrolysis perhaps by sequestering it away from PLC (Couchman *et al.* 2002, Kwon *et al.* 2009). However, this might be expected to attenuate rather than potentiate Ca^2+^ signaling, and no such activity has been reported for the other SDCs. Another possible mechanism of action for the enhanced Ca^2+^ signaling in the presence of SDCs could be through modulation of IP3R expression and/or activity. An investigation of the role of SDC1 on calcium mobilization in the vasculature suggests a modulatory role by this HSPG on IP3R function. Levels of phospho-IP3R in aortic rings from SDC1−/− mice are increased compared with those from wild-type mice (Potje *et al.* 2021). Based on this evidence, SDC1 could therefore be a positive modulator of IP3R function, since phosphorylation of IP3R can dampen its ability to mobilize Ca^2+^ (Kania *et al.* 2017). It thus remains to be determined how SDCs affect GHSR-induced Gα_q_ signaling cascades, and the potential implications of this mechanism for other GPCRs that signal through Gα_q_.

SDCs have been linked to receptor function by affecting several processes in cells. SDC2, for example, regulates the phosphorylation of the VEGF receptor (VEGFR2) by controlling the membrane localization of DEP1, a transmembrane phosphatase (Corti *et al.* 2022). SDC1 also regulates G-protein complex formation by mediating PECAM-1 binding to Gα_q_ in endothelial cells (dela Paz *et al.* 2014). Here, SDC1 is critical for the formation of the junctional complex and plays a role in the response of these junctions to flow rate through its heparan sulfate side chains. These heparan sulfate side chains also play a role in the allocation of ligands to receptors, such as that of the chemokine RANTES to CCR-5 (Slimani *et al.* 2003). While these are different systems or receptor types to energy homeostasis and GHSR, similar effects of SDCs have also been demonstrated in the hypothalamus. Here, SDC3 aids in the presentation of AgRP to MC4R, most likely through its heparan sulfate side chains (Creemers *et al.* 2006, Palomino *et al.* 2017, Chen *et al.* 2019). AgRP, an inverse agonist of MC4R, decreases the sensation of satiety. It is produced by AgRP/NPY neurons in the hypothalamus, which are activated by ghrelin stimulation. We speculate that SDCs may increase the neuronal response to ghrelin, leading to increased AgRP levels in the synaptic cleft. This effect is then further amplified by their facilitation of AgRP-binding to MC4R. Although this potential mechanism requires further investigation, GHSR (Zigman *et al.* 2005, Fernandez *et al.* 2018), ghrelin (Wortley *et al.* 2005) and SDC3 (Reizes *et al.* 2001, Strader *et al.* 2004) knockout mice share the phenotypes of having impaired reflex hyperphagia after fasting and resistance to diet-induced obesity. Intriguingly, global MRAP2 deletion in mice has the opposite, obesogenic, effect (Asai *et al.* 2013), despite blunting the orexigenic response to ghrelin (Srisai *et al.* 2017). However, like GHSR, ghrelin and SDC3 knockout mice, AgRP neuron-specific knockout of MRAP2 also impairs fasting-induced hyperphagia (Srisai *et al.* 2017). While we show that the overlap in expression between SDCs and GHSR is greater than that of MRAP2 and GHSR, it remains to be determined whether hypothalamic or AgRP-neuronal deletion of SDCs in mice has similar effects and whether SDCs and MRAP2 interact in their effects on GHSR signaling. Furthermore, it will be important to assess SDC and GHSR co-expression in neurons in greater detail using other techniques, such as spatial transcriptomics and RNAScope.

Altogether, we have identified SDCs as a new family of membrane proteins that affect GHSR signaling. The current data may provide an additional clue toward the mechanism by which SDCs play a role in eating behavior. Future work should focus on the physiological role of SDCs in ghrelin signaling and pinpoint the exact mechanism through which SDCs affect GHSR signaling and whether this mechanism also applies to other GPCRs.

## Declaration of interest

The authors declare that there is no conflict of interest that could be perceived as prejudicing the impartiality of the work reported.

## Funding

This research did not receive any specific grant from any funding agency in the public, commercial or not-for-profit sector.
